# Advancing Differentiation of Hepatic Metastases in Malignant Melanoma through Dual-Energy Computed Tomography Rho/Z Maps

**DOI:** 10.3390/diagnostics14070742

**Published:** 2024-03-30

**Authors:** Ibrahim Yel, Vitali Koch, Leon D. Gruenewald, Scherwin Mahmoudi, Leona S. Alizadeh, Aynur Goekduman, Katrin Eichler, Thomas J. Vogl, Mirela Dimitrova, Christian Booz

**Affiliations:** Clinic for Radiology and Nuclear Medicine, University Hospital Frankfurt, Goethe University Frankfurt, 60590 Frankfurt am Main, Germany; dr.ibrahimyel@gmail.com (I.Y.); vitali-koch@gmx.de (V.K.); gruenewald.leon@me.com (L.D.G.); scherwin.mahmoudi@gmail.com (S.M.); leona.alizadeh@outlook.de (L.S.A.); aynur.goekduman@med.uni-frankfurt.de (A.G.); k.eichler@em.uni-frankfurt.de (K.E.); t.vogl@em.uni-frankfurt.de (T.J.V.); boozchristian@gmail.com (C.B.)

**Keywords:** malignant melanoma, metastasis, dual-energy CT, Rho/Z, HU

## Abstract

Objectives: The aim of this study is to evaluate the diagnostic accuracy of dual-energy computed tomography (DECT)-based Rho/Z maps in differentiating between metastases and benign liver lesions in patients diagnosed with malignant melanoma compared to conventional CT value measurements. Methods: This retrospective study included 73 patients (mean age, 70 ± 13 years; 43 m/30 w) suffering from malignant melanoma who had undergone third-generation DECT as part of tumor staging between December 2017 and December 2021. For this study, we measured Rho (electron density) and Z (effective atomic number) values as well as Hounsfield units (HUs) in hypodense liver lesions. Values were compared, and diagnostic accuracy for differentiation was computed using receiver operating characteristic (ROC) curve analyses. Additional performed MRI or biopsies served as a standard of reference. Results: A total of 136 lesions (51 metastases, 71 cysts, and 14 hemangiomas) in contrast-enhanced DECT images were evaluated. The most notable discrepancy (*p* < 0.001) between measured values and the highest diagnostic accuracy for distinguishing melanoma metastases from benign cysts was observed for the Z (0.992; 95% CI, 0.956–1) parameters, followed by Rho (0.908; 95% CI, 0.842–0.953) and finally HU_120kV_ (0.829; 95% CI, 0.751–0.891). Conversely, when discriminating between liver metastases and hemangiomas, the HU_120kV_ parameters showed the most significant difference (*p* < 0.001) and yielded the highest values for diagnostic accuracy (0.859; 95% CI, 0.740–0.937), followed by the Z parameters (0.790; 95% CI, 0.681–0.876) and finally the Rho values (0.621; 95% CI, 0.501–0.730). Conclusions: Rho and Z measurements derived from DECT allow for improved differentiation of liver metastases and benign liver cysts in patients with malignant melanoma compared to conventional CT value measurements. In contrast, in differentiation between liver hemangiomas and metastases, Rho/Z maps show inferior diagnostic accuracy. Therefore, differentiation between these two lesions remains a challenge for CT imaging.

## 1. Introduction

Cutaneous malignant melanoma, while representing only 4% of all skin tumors, is responsible for approximately 80% of all skin cancer deaths. It represents one of the most aggressive and dangerous skin tumors and is often associated with a poor prognosis [1]. The incidence is increasing, especially in fair-skinned people, on a global scale. Australia has the highest melanoma incidence rates worldwide. In Europe, the melanoma incidence is highest in the northern regions and lowest in the southern regions [2]. In 2020, 325,000 new cases of melanoma were estimated globally, while 57,000 people died of the disease [3]. The number of new cases in Germany has increased more than fivefold in the last 50 years [4]. Nowadays, malignant melanoma is considered to be a multi-factorial disease. In evaluating risk factors, exogenous and endogenous factors are distinguished. The most crucial external melanoma risk is increased ultraviolet exposure, especially in early childhood [5]. In adults, both artificial and natural UV radiation increase the risk of developing melanoma. Even when compared, spending time in a solarium is significantly more dangerous than bathing outside in the sun when it comes to developing skin cancer. The average age of diagnosis is 60 years for women and 68 years for men. Furthermore, malignant melanoma can occur on any part of the body. In women, malignant melanoma is most commonly found on the lower legs, whereas in men, the head and trunk are most commonly affected.

Malignant melanoma in the advanced, metastatic stage almost always has a lethal outcome with a short survival time. Therefore, early and highly accurate tumor staging is crucial for treatment decisions and prognosis [6]. Current guidelines recommend staging CT scans of the chest and abdomen as a standard imaging modality due to its widespread availability [7,8]. CT imaging, while instrumental in diagnosing various medical conditions, often encounters challenges in confidently distinguishing between malignant and benign lesions. One of the primary limitations lies in the inherent nature of CT scans, which primarily provide anatomical information with limited tissue characterization capabilities. Consequently, subtle differences in the imaging characteristics of malignant and benign lesions may not always be discernible with CT images alone. Additionally, certain benign lesions can manifest with features that mimic malignancy, further complicating the diagnostic process. Moreover, the reliance on morphological criteria, such as lesion size and enhancement patterns, for differentiation may lead to inaccuracies, as these features can overlap between benign and malignant entities. For instance, cysts, hemangiomas, and metastases may share similar CT values due to factors like low vascularity and necrosis within metastatic lesions, contributing to diagnostic uncertainty [9,10]. Moreover, it is worth noting that liver metastases may occasionally present with high attenuation due to hemorrhagic content, which can pose challenges in differentiating them from hemangiomas [11]. The differentiation of cysts can also pose challenges, especially when presenting with septations, nodularities, or hemorrhagic contents. In addition, small, hypodense metastases may be difficult to differentiate from benign hepatic lesions. Small lesions (<1 cm) are rarely malignant in patients without a known primary disease. However, it is important to note that in patients with known primary tumors, lesions meeting this criterion have up to a 30% chance of being malignant [12,13]. As a result, CT imaging may necessitate supplementary diagnostic modalities or clinical correlation to enhance diagnostic confidence in distinguishing between the malignant and the benign. MRI or biopsies are often for used for accurate differentiation. Nevertheless, limited MRI availability, specific contraindications, and the invasive character of biopsies with potential complication risks are critical in some patients. Furthermore, compared to computed tomography, MRI examinations are also significantly more complex, time-consuming, and associated with higher costs. As previously noted, CT imaging has its limitations. In this study, our primary focus lies in enhancing the capabilities of CT imaging, specifically aiming to overcome some of these limitations.

Over the past few years, technological advancements in CT have improved image quality, reduced scan times, and even provided additional information to improve diagnostic accuracy. In this context, dual-energy CT (DECT) has been proven to be a highly accurate method for tumor assessment due to its shown improved material characterization and differentiation compared to conventional single-energy CT [14,15]. In this context, DECT postprocessing provides a variety of additional quantitative parameters of tumor characteristics, including atomic number maps (Rho/Z) showing the Rho and Z of lesions [16,17]. Numerous studies have assessed DECT advantages, especially in oncology [14,18,19,20,21,22]. However, this technique has not been evaluated to differentiate liver metastases and benign lesions in patients with malignant melanoma. Therefore, this study aimed to investigate and compare the diagnostic accuracy of DECT-based Rho and Z values and conventional HU measurements for the differentiation of MRI- or biopsy-proven hypodense liver metastases and benign lesions in patients suffering from malignant melanoma.

Until now, hypodense liver lesions in patients with malignant melanoma could only be detected in computed tomographic staging using image morphological elements. With the dual-energy CT technology routinely used at our institute in recent years, better material differentiation is now possible due to the tube voltage discrepancies in the two X-ray tubes. The aim of this work is to improve the detection of malignant melanoma metastases in the liver using dual-energy computed tomography in combination with special computer software. It was investigated to what extent patients with diagnosed malignant melanoma can be distinguished between benign liver lesions and liver metastases on the basis of electron density and effective atomic numbers. This could result in an additional method of CT diagnosis, of metastases of malignant melanoma, to HU measurement.

## 2. Materials and Methods

The institutional review board approved this retrospective study. The requirement to obtain written informed consent was waived.

### 2.1. Study Population

**Figure 1:**
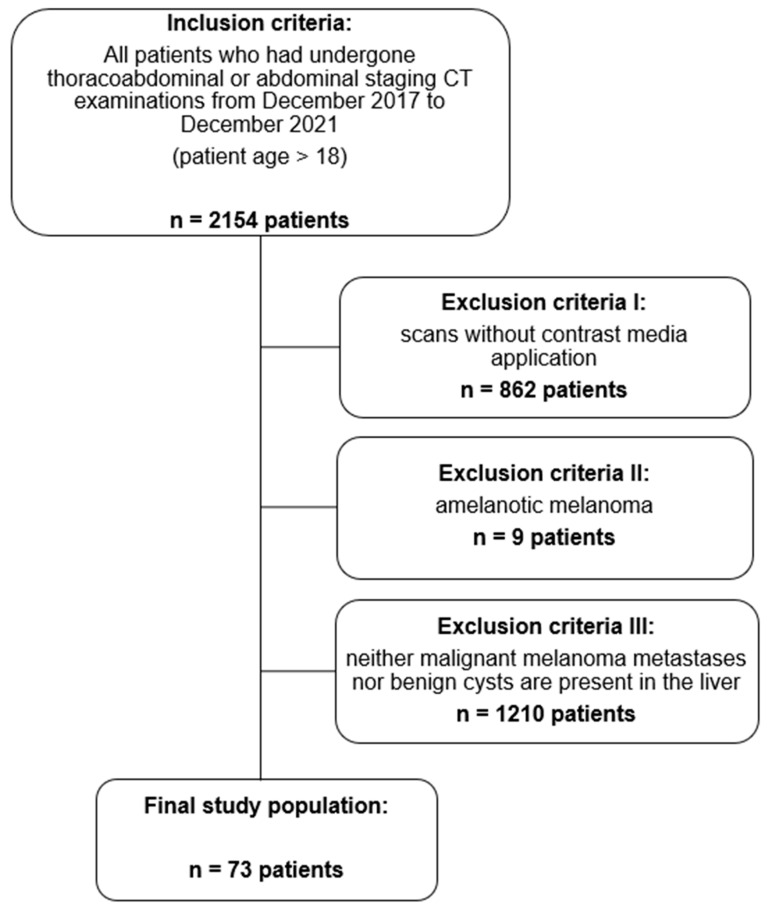
Flow chart showing the selection process in this study.

A total of 2154 patients (patient age > 18 years) with histologically confirmed malignant melanoma who had undergone routine third-generation dual-source DECT malignant melanoma staging between December 2017 and December 2021 were considered for inclusion in this retrospective study. The exclusion criteria were amelanotic melanoma (*n* = 9 patients), scans without contrast media application (*n* = 862 patients), and patients with neither malignant melanoma metastases nor benign cysts in the liver (*n* = 1210 patients). The final study population consisted of 73 patients. Figure 1 illustrates the patient selection process in this study.

### 2.2. Dual-Energy CT Scan Protocol

Routine chest–abdominal staging CT scans were performed with third-generation dual-source DECT (Somatom Force, Siemens Healthineers, Forchheim, Germany) with the intravenous administration of a contrast agent after an 85 s delay. Contrast media (Imeron 400 mgI/mL, Bracco, Milan, Italy) were intravenously injected at a dose of 1.3 mL per kilogram of body weight and a flow rate of 3 mL/s through a superficial vein of the forearm. The CT examinations were all performed in the craniocaudal scan direction using DE mode, in which two X-ray tubes were operated at two different voltage levels (tube A: 100 kV and tube B: Sn150 kV with a tin filter). The rotation time was 0.5 s, collimation width 128 × 0.6 mm, and pitch 0.6 mm.

### 2.3. CT Image Reconstruction

In each CT scan, three different image sets were acquired, 100 kVp, Sn150 kVp, and the calculated weighted average (ratio 0.5:0.5), to resemble the image properties of a single-energy 120 kVp scan [21]. Standard reconstructions (axial, coronal, and sagittal; section thickness, 1 mm; increment, 0.75 mm) were generated using a dual-energy medium-soft convolution kernel (Qr40, advanced model-based iterative reconstruction [ADMIRE] level of 3) for the high- and low-kilovolt series. All reconstructions were transferred to the picture archiving and communication system (PACS) for image evaluation.

### 2.4. CT Measurements

DECT image series were postprocessed on a dedicated DECT workstation (syngo.via, version VB10B; Siemens Healthineers, Erlangen, Germany) using the Rho/z map algorithm to achieve tissue differentiation based on Rho and Z. The calculation parameters of the generated data were selected as follows: resolution, 10; minimum HU_120kV_ threshold, 40; maximum HU_120kV_ threshold, 50.

Circular regions of interest (ROIs) were placed in malignant melanoma liver metastases and liver cysts to obtain Rho/z and HU_120kV_ data, avoiding lesion margins, large blood vessels, and surrounding artifacts. Each lesion was measured ten times, and the average was calculated.

### 2.5. Reference Standard

MRI or biopsy served as a standard reference in this study for lesion definition. The MRI scan protocols included T1-weighted imaging before and after intravenous injection, T2-weighted sequences with and without fat saturation, diffusion-weighted images, and corresponding apparent diffusion coefficient (ADC) maps. MRI interpretation, as well as the identification of lesions in cases where biopsy served as reference, were performed by one board-certified radiologist (blinded) with 31 years of experience in liver imaging.

### 2.6. Statistical Analysis

Statistical analysis was performed using commercially available software (MedCalc for Windows, version 13, and GraphPad Prism for Windows, version 7). The Kolmogorov–Smirnov test was used for analyzing the normality of data. Variables were given as means ± standard deviations and analyzed with the Wilcoxon matched-pairs test. *p* < 0.05 was considered to show a statistically significant difference.

For better visualization of the data variance, we recalculated the mean differences (MDs) between melanoma liver metastases and benign lesions into percentage differences, as the Z values were measured with a different unit than Rho and HU_120kV_. The calculations were made with the following equation:

$$Percentage\;difference=\frac{{MD}_{metastases}-{MD}_{benign\;lesion}}{({MD}_{metastases}+{MD}_{benign\;lesion})/2}\times 100$$

For the quantitative image analysis, ROC curve analysis and the area under the curve (AUC) were applied to define optimal cut-off values for the differentiation of liver lesions. For these optimal cut-off values, sensitivity and specificity values were calculated. The values of overall sensitivity, specificity, positive predictive value (PPV), and negative predictive value (NPV) were given as means. Finally, AUC values were compared to demonstrate significant differences and to calculate the standard error using the DeLong test.

## 3. Results

A total of 136 lesions (51 metastases, 38%; 71 cysts, 52%; and 14 hemangiomas, 10%) were evaluated in 73 patients (70 ± 13 years; range, 39–92) consisting of 30 women (41%; 69 ± 15 years; range, 39–90) and 43 men (59%; 70 ± 12 years; range, 47–92). An average of one lesion per patient was reported, ranging from one to three. The characteristics of the study population are presented in Table 1.

The Z and Rho values showed a significant difference (*p* < 0.001) between malignant melanoma metastases and benign liver cysts (Z, MD, 1.613 ± 0.0921; percentage difference (PD), 110%; and Rho, MD, 34.71 ± 3.318 and PD, 88%). The HU_120kV_ measurements (MD, 22.46 ± 3.007; PD, 63%) also demonstrated a significant difference (*p* < 0.001) for metastases and cysts. However, the Z parameters showed the greatest difference in measured values, followed by Rho and finally HU_120kV_. When comparing hemangiomas and metastases, the most pronounced dissimilarity (*p* < 0.001) was observed in the HU_120kV_ values (MD, 21.62 ± 7.45; PD, 37%), with secondary distinctions noted in the Z (MD, 0.60 ± 0.12; PD, 23%) and Rho (MD, 11.27 ± 5.12, PD, 21%) parameters. The quantitative parameters for the Rho, Z, and HU_120kV_ parameters are summarized in Table 2.

**Figure 2:**
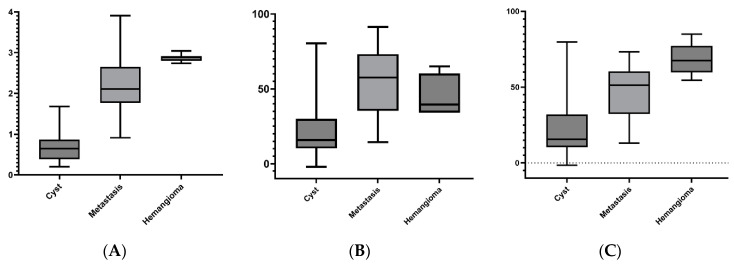
Box-and-whisker plots visualize the comparison of region-based mean Z values (**A**), Rho values (**B**), and CT numbers for 120 kV in HUs (**C**) between benign cysts, malignant melanoma metastases, and hemangiomas. Overall, the most significant disparity between cysts and metastases is observed in the Z and Rho values, whereas for hemangiomas and metastases, the most significant differentiation is observed in the HU_120kV_ parameters, followed by the Z values.

To visualize the measurement differences between benign lesions and metastases, box-and-whisker plots (Figure 2) are used to show the data distribution through their quartiles.

The ROC curve demonstrated that the Rho and Z parameters have high diagnostic accuracy for differentiating malignant melanoma liver metastases and benign cysts. The Z values indicated the highest AUC value (0.992; 95% CI, 0.956–1), followed by Rho (0.908; 95% CI, 0.842–0.953). In both measurements, the sensitivity ranged from 98.04% (95% CI, 89.6–100) for Z and 96.08% (95% CI, 86.5–99.5) for Rho, while the specificity was 95.77 (95% CI, 88.1–99.1) for Z and 74.65% (95% CI, 62.9–84.2) for Rho. In comparison, the HU_120kV_ parameters showed a lower AUC value (0.829; 95% CI, 0.751–0.891) as well as lower sensitivity (86.27%; 95% CI, 73.7–94.3) and specificity (63.38%; 95% CI, 51.1–74.5).

In a comparative analysis, the ROC curve for distinguishing hemangioma versus metastasis exhibited the highest AUC value with the HU_120kV_ parameters (0.859; 95% CI, 0.740–0.937), followed by the Z parameters (0.790; 95% CI, 0.681–0.876). Conversely, the Rho parameters demonstrated the lowest AUC value in this context (0.621; 95% CI, 0.501–0.730). Notably, the Z and Rho parameters (Z, 95.83%; 95% CI, 78.9–99.9 and Rho, 95.83%; 95% CI, 78.9–99.9) demonstrated superior specificity in comparison to the HU_120kV_ parameters (80%; 95% CI, 28.40–99.50). The AUC values, along with their corresponding 95% CI, sensitivities, specificities, PPVs, and NPVs for each lesion and parameter, are presented in Table 3 and Table 4.

**Figure 3:**
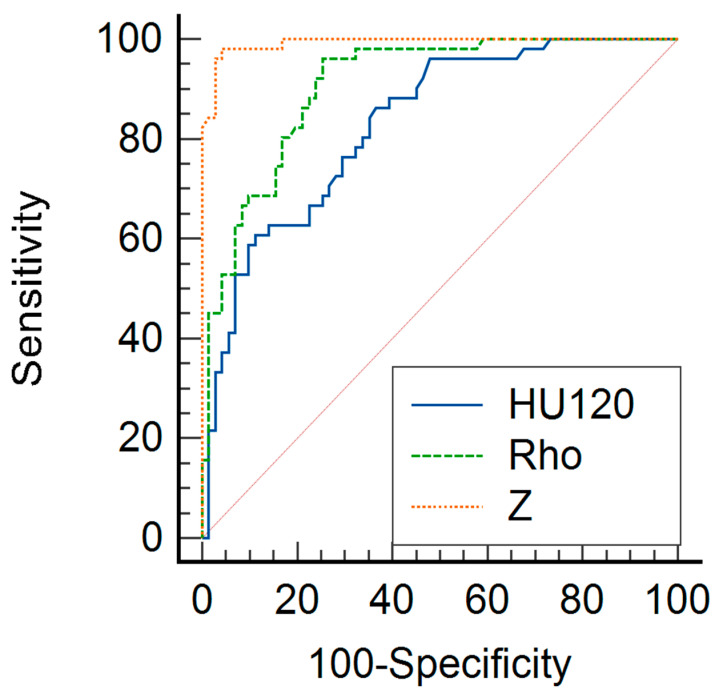
Comparison of representative ROC curves of Z values (orange line), Rho values (green line), and CT numbers for 120 kV in HUs (blue line) for differentiation between benign cysts and malignant melanoma metastases. The Z (area under the curve [AUC] = 0.992) and Rho (AUC = 0.908) parameters yielded significantly higher diagnostic accuracy for the differentiation between cysts and metastases compared to the HU_120kV_ measurements (AUC = 0.829).

**Figure 4:**
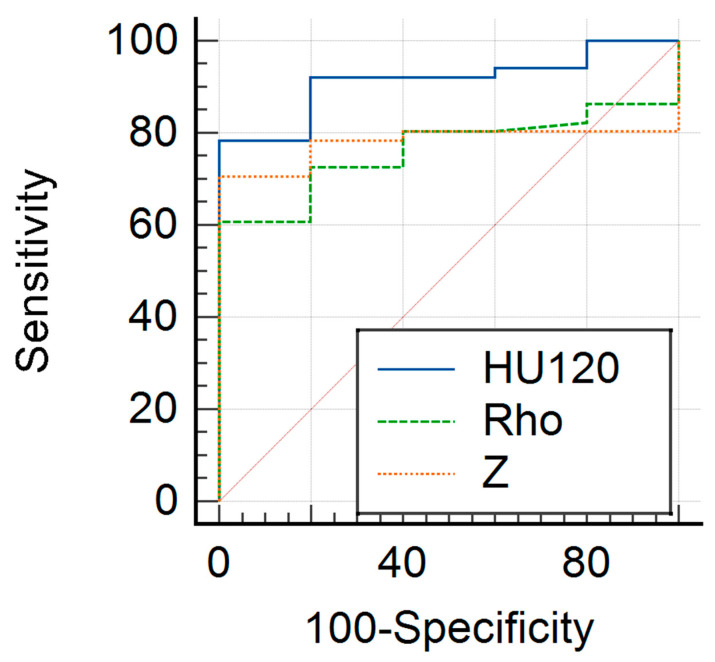
Comparison of representative ROC curves of Z values (orange line), Rho values (green line), and HU_120kV_ parameters (blue line) for differentiation between hemangioma and malignant melanoma metastases. The HU_120kV_ measurements (AUC = 0.859) and Z (AUC = 0.790) parameters yielded significantly higher diagnostic accuracy for the differentiation between hemangioma and metastases compared to Rho (AUC = 0.621).

The diagnostic performances of Rho, Z, and HU_120kV_ in differentiating liver lesions are displayed as ROC curves in Figure 3 and Figure 4.

**Figure 5:**
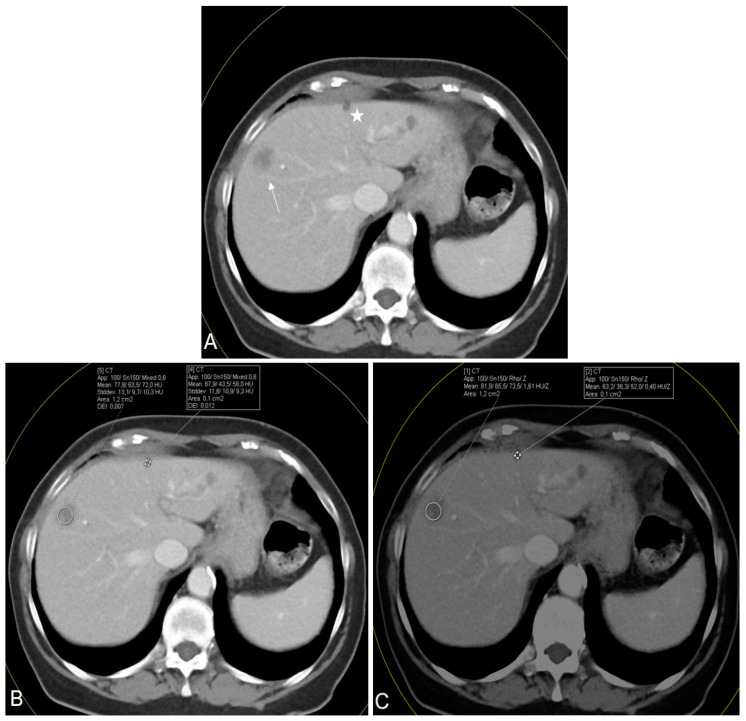
Axial contrast-enhanced CT scan in a 54-year-old female suffering from malignant melanoma. The scan showed multiple hypodense liver lesions in different segments, including a biopsy-confirmed liver metastasis in segment 8 (arrow) and an MRI-confirmed subcapsular liver cyst in segment 4 (star) (**A**). Conventional CT value measurements (**B**) demonstrated surprisingly high mean CT values for both lesions (72.0 and 58.0, respectively), while the mean Rho and Z values (**C**) showed greater differences for both lesions (Rho, 79.5 and 51.9; Z, 1.53 and 0.40, respectively) facilitating CT-based differentiation between metastases and cysts.

In Figure 5, an exemplary CT scan of a patient reveals multiple hypodense liver lesions in the context of malignant melanoma. Despite the acquisition of HU measurements, distinguishing between these lesions proved challenging. Nevertheless, a clear diagnosis remained challenging, which is why Rho/Z maps were derived.

## 4. Discussion

The results of this retrospective study demonstrate the improved diagnostic accuracy of Rho/Z maps and corresponding measurements reconstructed from DECT data in differentiating malignant from benign liver lesions in patients with malignant melanoma compared to conventional CT value measurements. In summary, when discriminating between metastases and cysts, the Z values (AUC, 0.992; 95% CI, 0.956–1) and Rho values (AUC, 0.908; 95% CI, 0.842–0.953) showed superior diagnostic accuracy measures than the HU_120kV_ parameters (AUC, 0.829; 95% CI, 0.751–0.891), indicating the beneficial potential of DECT in assessing hypodense liver lesions in patients with malignant melanoma undergoing staging CT examinations. Conversely, when discriminating between metastases and hemangiomas, the HU_120kV_ parameters yielded higher diagnostic accuracy values (0.859; 95% CI, 0.740–0.937), followed by the Z values (0.790; 95% CI, 0.681–0.876) and finally the Rho values (0.621; 95% CI, 0.501–0.730). However, the Rho and Z parameters (Rho, 95.83%; 95% CI, 78.90–99-9 and Z, 95.83%; 95% CI, 78.90–99.9) exhibited higher specificity compared to HU_120kV_ (80%; 95% CI, 28.40–99.50), indicating that while differentiation between metastases and hemangiomas in CT scans remains challenging when relying solely on visual characteristics and standard HU values, the use of Rho/Z maps can enhance diagnostic confidence in differentiating between liver metastases and benign cysts. This is crucial for patient outcomes because metastases are the leading cause of death associated with melanoma, with a 5-year survival rate of 23% for patients with metastases at the time of diagnosis [23]. The extent of the disease and the location of distant metastases determine what kind of therapy is required. Generally, as soon as distant metastases occur, therapy is only palliative. However, recent studies have shown that targeted immunotherapies can prolong survival. Nevertheless, the outcome of the treatment is dependent heavily on a patient’s immunological status and the stage of the tumor [23,24]. In this context, numerous patients present with incidental benign liver lesions, with cysts being the most prevalent. Given the potential consequences of misdiagnosing a benign liver lesion as a metastasis, avoiding inappropriate treatment decisions is paramount. Therefore, achieving highly accurate staging that includes the liver is essential to promptly initiate optimal treatment and ultimately improve patient outcomes.

CT-based differentiation between liver lesions remains a challenge. The appearance of liver metastases in CT images may vary based on factors such as blood supply, hemorrhage, cellular differentiation, fibrosis, and necrosis, posing challenges in differentiation, particularly from hemangiomas. In addition, simple benign cysts can become complex if they are infected, hemorrhaged, or ruptured, increasing the HU value above the average value (0–20 HUs). Consequently, benign liver cysts may exhibit HU values similar to liver metastases in certain instances [25,26]. Furthermore, small lesions (<1 cm) pose difficulties in diagnosis. Therefore, accurate assessment of these liver lesions often necessitates biopsy or additional MRI evaluation [27,28]. However, limited MRI availability, specific contraindications, and the invasive nature of biopsy may introduce potential complications for some patients. As a result, staging melanoma patients can be particularly challenging in certain scenarios.

In recent years, DECT has become one of the main focuses of interest in CT-based oncological imaging owing to its many advantages, including better material characterization and differentiation. DECT is based on the principle that the attenuation of X-rays in tissue (expressed as the CT attenuation number in HUs) depends on the tissue density but also on the Z of the specific tissue and on the energy of the photon beam. Thus, DECT can thereby also quantify lesion iodine content. Various postprocessing techniques provide additional information to distinguish lesions by analyzing these parameters. Former studies have demonstrated DECT’s advantage in distinguishing benign lesions from malignant ones compared to conventional single-energy CT [14,18,29,30,31]. However, there are insufficient studies that performed multiparametric analysis based on the Z and Rho of each tissue type using the application class of special postprocessing software (Rho/Z maps), especially in patients with malignant melanoma.

Different postprocessing techniques on dedicated software, for example, syngo.via (our software of choice), provide additional information for more accurate diagnosis of lesions by analyzing their electron density, effective atomic number, and iodine concentration. There have been a few previous studies that have shown the value of Rho/Z maps as an accurate addition to CT oncological diagnostics. Mileto et al. have shown that non-enhancing renal cysts, including hyperattenuating cysts, can be distinguished from enhancing masses on effective atomic number maps derived from dual-energy CT. In this study, the analysis showed an AUC for Z of greater than 0.9 (0.92; 95% CI, 0.89–0.94) for the evaluation of renal masses, indicating high diagnostic accuracy [32]. DECT-based Rho/Z maps were also used by Chijie Xu et al. to better distinguish osteoblastic metastases from bone islands (AUC for Z, 0.91; AUC for Rho, 0.88). Our findings are in accordance with the studies by Mileto et al. and Chijie Xu et al. and emphasize the value of DECT in abdominal oncologic imaging. Further studies have shown similar results for DECT Rho/Z measurements for other body regions, such as head and neck imaging, when differentiating benign from malignant thyroid nodules and T1 stage nasopharyngeal carcinoma from benign hyperplasia [33,34].

Current diagnostic techniques for identifying liver lesions and staging in melanoma patients involve a multifaceted approach utilizing various imaging modalities. Ultrasound, a widely accessible, non-invasive, and inexpensive tool, serves as an initial screening method due to its ability to detect hepatic lesions. However, its effectiveness may be limited by factors such as operator skill, patient cooperation, and the presence of bowel gas interference [35]. PET-CT has emerged as a key diagnostic tool for detecting liver lesions. This imaging modality combines the functional information obtained from PET, which highlights metabolic activity within tissues, with the anatomical details provided by CT imaging. PET-CT offers several advantages in the evaluation of liver lesions, including its ability to detect lesions with high metabolic activity indicative of malignancy, thereby aiding in the differentiation between benign and malignant lesions. However, despite its utility, PET-CT does have limitations. Notably, it presents a comparatively lower spatial resolution compared to MRI, potentially impacting the delineation of fine anatomical details. Additionally, concerns regarding accessibility and ionizing radiation exposure persist, with PET-CT’s availability being less widespread than other imaging modalities and its utilization carrying a heightened risk of radiation exposure. MRI presents another crucial modality in liver lesion diagnostics, with superior soft tissue contrast resolution. Its capacity to detect nuanced alterations in liver tissue renders it highly adept at identifying small lesions and characterizing tumors. However, MRI does present limitations, primarily its lower availability compared to other imaging modalities. Furthermore, challenges arise in cases of patient non-cooperation, potentially leading to suboptimal study outcomes. Additionally, MRI may be contraindicated in patients with metal implants or those experiencing claustrophobia [36]. In contrast, DECT data facilitate rapid reconstruction of Rho/Z maps, with measurements easily accessible, making it a time-efficient and valuable additional clinical tool. This postprocessing tool is particularly beneficial for patients with MRI contraindications or coagulopathies prohibiting biopsy. Additionally, CT scans are readily available during on-call periods, unlike MRI or biopsy, potentially expediting diagnosis and treatment in specific cases. This highlights the potential of DECT-based Rho/Z maps as a versatile and efficient tool in clinical practice, particularly for patients with limitations to other imaging modalities or invasive procedures.

This study has limitations that need to be discussed. First, the present study is a single-center retrospective study, which may limit the generalizability of its findings. Second, our research was limited to a vendor-specific CT system and may not be applicable to other DECT technologies. Third, the scope of our analysis was limited to benign cysts, hemangiomas, and metastases in the liver, which are indeed the predominant liver lesions encountered in clinical practice. Further, our study is limited by a relatively small sample size of only 73 patients. Increasing the sample size would provide a broader understanding of our research and strengthen the reliability of our findings. Additionally, the limited number of participants may impede the detection of subtle yet clinically significant associations or effects within the patient population. Moving forward, it is essential for subsequent research efforts to replicate our study in larger cohorts to ensure the validity and generalizability of our findings. Nevertheless, future studies should aim to investigate the extent to which the Rho/Z values of cysts, hemangiomas, and metastases differ from other liver lesions, thus providing deeper insights into lesion differentiation. In addition, it should be noted that our research focused primarily on contrast-enhanced CT images. Therefore, there is a need for future investigations to assess the utility of non-contrast DECT images in this context. Further, the results of this study are specific to melanoma metastases and cannot be generalized to staging studies in the settings of other cancers.

Another limitation in the present study is that the impact of DECT on clinical outcomes was not investigated. While our study focused on evaluating the diagnostic accuracy of DECT-derived parameters in distinguishing between liver metastases and benign liver lesions in patients with melanoma, the broader implications of DECT on patient management and treatment outcomes were not explored. Further research is needed to assess the potential clinical benefits of DECT in influencing patient outcomes and guiding therapeutic decisions. Nonetheless, our findings suggest the potential applicability of Rho/Z maps to other malignancies featuring liver metastases and benign liver lesions, thereby warranting further investigation.

## 5. Conclusions

In conclusion, DECT-based Rho and Z measurements offer enhanced differentiation between liver metastases and benign liver cysts in patients with malignant melanoma compared to conventional CT measurements. Improved detection and characterization of lesions by DECT could expedite the diagnostic process and improve staging accuracy in patients with malignant melanoma, a critical factor for guiding treatment decisions. Nevertheless, patients with contraindications for other imaging modalities and diagnostic methods may particularly benefit from DECT-based Rho and Z measurements. This highlights the importance of considering DECT as a valuable additional tool in the staging of liver metastases in patients with malignant melanoma. Thus, if technically possible, Rho/Z maps and corresponding measurements should be applied in the context of tumor staging in patients with malignant melanoma and the presence of hypodense liver lesions.

In contrast, in the differentiation between hemangiomas and liver metastases, Rho/Z maps show inferior diagnostic accuracy compared to HU measurements. Therefore, differentiation between these two lesion types remains a challenge in CT imaging.

## Figures and Tables

**Table 1:** Characterization of the study population (*n* = 73).

Characteristics	Value
Number of patients	73
Mean age ± SD, range	70 ± 13, 39–92
Men	43/73 (0.59)
Mean age of men ± SD, range	70 ± 12, 47–92
Women	30/79 (0.41)
Mean age of women ± SD, range	69 ± 15, 39–90
Average number of lesions per patient, range	1, 1–3

**Table 2:** Rho/Z and HU_120kV_ mean values for malignant melanoma metastasis, cysts, and hemangiomas.

Parameters	Cyst (*n* = 71)	Metastasis (*n* = 51)	Hemangioma (*n* = 14)
Rho	21.89 ± 16.24	56.60 ± 20.36	45.43 ± 12.76
Z	0.63 ± 0.33	2.26 ± 0.96	2.87 ± 0.30
HU_120kV_	24.27 ± 1.957	46.73 ± 16.23	68.35 ± 10.87

**Table 3:** Diagnostic accuracy of Rho, Z, and HU_120kV_ in differentiating benign liver cysts and melanoma metastases.

Parameters	AUC	Sensitivity (%)	Specificity (%)	PPV (%)	NPV (%)
Rho	0.908	96.08	74.65	73.1	96.4
	(0.842–0.953)	(86.5–99.50)	(62.9–84.20)	(64.5–80.30)	(87.1–99)
Z	0.992	98.04	95.77	94.3	98.6
	(0.956–1)	(89.6–100)	(88.1–99.1)	(84.6–98.10)	(90.7–99.80)
HU_120kV_	0.829	86.27	63.38	62.9	86.5
	(0.751–0.891)	(73.7–94.30)	(51.1–74.50)	(55–70.10)	(76–92.90)

**Table 4:** Diagnostic accuracy of Rho, Z, and HU_120kV_ in differentiating liver hemangiomas and melanoma metastases.

Parameters	AUC	Sensitivity (%)	Specificity (%)	PPV (%)	NPV (%)
Rho	0.621	39.20	95.83	95.2	42.6
	(0.501–0.730)	(25.8–53.9)	(78.9–99.9)	(74.0–99.3)	(37.0–48.4)
Z	0.790	70.59	95.83	97.30	60.5
	(0.681–0.876)	(56.2–82.5)	(78.9–99.9)	(84.0–99.6)	(49.9–70.3)
HU_120kV_	0.859	82.35	80.00	91.1	61.5
	(0.740–0.937)	(69.1–91.6)	(28.4–99.5)	(80.9–96.1)	(46.8–74.4)

## Data Availability

The data presented in this study are available on request from the corresponding author The data are not publicly available due to data protection.

## Abbreviations

ADC	Apparent diffusion coefficient
AUC	Area under the curve
DECT	Dual-energy CT
MD	Mean difference
NPV	Negative predictive value
PACS	Picture archiving and communication system
PPV	Positive predictive value
Rho	Electron density
ROI	Circular regions of interest
ROC	Receiver operating characteristic
SD	Standard deviation
CI	Confidence interval
Z	Effective atomic number
